# Fasting

**Published:** 1890-04

**Authors:** 


### FASTING.

Carnivorous animals, according to M. Charles Richet, bear fasting better than the herbivorous, the latter being accustomed to eat almost continuously instead of at uncertain intervals. Dogs can endure abstinence 30 days on the average, and cold blooded animals twice that time. A python, however, has been known to live 23 months without eating, a rattlesnake 29 months, a tortoise 18 months and a frog 16 months. The endurance depends greatly upon the activity of the nervous system, which is wonderfully reduced during the torpidity of hibernating creatures, but the fatal limit of fasting is usually reached in all animals when the loss in weight reaches 40 per cent. In the case of man, in health, the estimate seems justified that 20 days of fast will bring death. But with special preparations, as in the cases of Tanner, Succi and Merlatti, fasting seems to have been prolonged to twice this period, or longer, without fatal result. Diseased persons often live much longer without food than healthy ones could. A Dutch hysteric woman died in 1826 after nearly four years of fasting; and another woman died in 1828 after a lethargic fast of six months. Many cases of wonderful fasts, less entitled to belief, are given by medical writers of two or three centuries ago. Among them is that of a girl of Heidelberg who neither ate nor drank for seven years, that of a girl of Spires who lived for three years upon nothing but a few drops of water or wine, and that of a girl of Cologne who fasted four years and fainted at the taste of food. In the last century a woman is said to have lived 26 years in a state of partial fasting relieved at intervals by a few drops of milk or broth; and another woman ate in a year only what an ordinary person would require for two days.

